# Supplementary material for: Fabricating Pea Protein Micro-Gel-Stabilized Pickering Emulsion as Saturated Fat Replacement in Ice Cream
Source: Foods. 2024 May 13;13(10):1511. doi: 10.3390/foods13101511 (PMC11121546; doi:10.3390/foods13101511)
Supplement: Supplementary file 1 [file foods-13-01511-s001.zip › foods-2985301-supplementary.pdf]

Table S1 Stock solutions preparation of simulated digestion fluids

		SSF	SGF	SIF
Constituent	Concentration(mol/L)	Volume(mL)	Volume(mL)	Volume(mL)
KCl	0.5	15.1	6.9	6.8
KH_2PO_4	0.5	3.7	0.9	0.8
$NaHCO_3$	1	6.8	12.5	42.5
NaCl	2	/	11.8	9.6
$MgCl_2$•$(H_2O)_6$	0.15	0.5	0.4	1.1
$(NH_4)_2CO_3$	0.5	0.06	0.5	/
Adjust pH for		pH7.0	pH3.0	pH7.0
Add water to make up to 500 mL				
$CaCl_2$•$(H_2O)_2$*	0.3	2.5	0.25	1

*$CaCl_2$•$(H_2O)_2$ was subjected to the mixture of simulated digestion fluids after digestive enzymes was added.
